# A Scoping Review of the Use of Improvised External Ventricular Drains in Africa

**DOI:** 10.7759/cureus.28748

**Published:** 2022-09-03

**Authors:** Damilola Jesuyajolu, Gamaliel Aremu, Olatomiwa Olukoya, Kennedy Obiekwe, Charles Okeke, Emmanuel Edeh, Terngu Moti, Abdulahi Zubair

**Affiliations:** 1 Neurosurgery, Surgery Interest Group of Africa, Lagos, NGA

**Keywords:** external ventricular drain (evd), catheter, africa, hydrocephalus, drains

## Abstract

Poor access to neurosurgical equipment is one of the problems limiting service delivery in Africa. Improvised surgical devices have long been used in Africa as replacements for high-cost standard versions. In this study, we aimed to see if improvised external ventricular drains (EVD) are being used, how these devices are made, and what their outcomes are. The PRISMA extension for scoping reviews was used in conducting this study. A search was conducted from inception to July 2022. PubMed, Ovid Embase, and African Journal Online were searched. Three studies were identified and used. The methods of making the EVD devices were compared and the incidence proportions of improvised EVD-related infections were calculated. The standard ventricular catheter was replaced by cheaper alternatives like a size 6/8 feeding tube or a 14-gauge central line catheter. The connecting tube had low-cost alternatives, and in a study, was replaced by a fluid infusion set. Aggregated outcomes from the three identified studies show that just over half of the sample survived post-EVD insertion (54%). The incidence proportion of EVD-related infections was 24%. This study describes the experience of African centers with an improvised version of the EVD devices and their outcomes. This will serve as a baseline for more research into the use of improvised EVD devices in low-resource settings.

## Introduction and background

Poor access to neuroimaging, operating microscopes, facilities, and surgical consumables are some of the problems faced by the already limited number of neurosurgeons in Africa [1,2]. These problems often cause substantial impairment in the quality of service rendered when compared to developed countries. A recent study involving neurosurgeons cited poor access to neurosurgical equipment as one of the problems limiting service delivery in African countries [3]. Improvised surgical devices have long been used in Africa as a replacement for high-cost standard versions. Thoracostomy, feeding gastrostomy, tourniquet, drains, and silos are procedures that have utilized improvised materials [4].

External ventricular drains (EVDs) are devices used to aid the removal of cerebrospinal fluid and monitoring of pressure in patients with raised intracranial pressure [5]. Common indications for its placement include management of hydrocephalus, intracranial pressure management in traumatic brain injury, and CSF diversion for collections of CSF or leaks [6-8]. EV drainage involves passing a catheter into the lateral ventricle and connecting it through a system of tubes to a collection bag at a prescribed height. This can be performed by drilling a burr hole, which is done in a theater, or with the use of a twist drill and a hollow bolt done at the bedside [9]. The EVD device is made up of different parts. These include the ventricular catheter, proximal connecting tube, proximal collecting system/three-way stopcock, connector tube to main EVD system, stopcock, adjustable EVD set, graduated burette for monitoring CSF flow, another stopcock, a connecting tube, and a collection bag [10]. Some devices have been modified to also measure intracranial pressure [11].

The cost of EVD placement has been quoted in literature to range between $1300 and $3200 [12]. The cost of the kit itself, if placed with a bolt, has been quoted to cost between $303 and $1547 [13]. In Africa, where the average income is less than $800 and payment is made out-of-pocket due to poor insurance coverage, it is clear that this device is expensive and not readily available at every institution with supply chain limitations. There is a need for a low-cost alternative that will be available at most institutions providing neurosurgery care in Africa. If these devices could be improvised and used in a sterile manner, the quality of neurosurgical care given in low-resource settings could improve dramatically. It is against this background that we aimed to examine the existing literature to see if improvised devices are being used and to see how these devices are made. Furthermore, we aimed to see the outcomes following the insertion of these improvised devices, in terms of the incidence proportion of EVD-related infections.

## Review

Methodology

The PRISMA extension for scoping reviews was used in conducting this study. Papers that considered improvised EV drainage systems, their usage, method of construction, and outcomes in African surgical centers were included in this study. Studies that were not in the English language, from non-African countries, and studies that did not consider EVD systems were excluded. Reviews, meta-analyses, abstracts, conference presentations, commentaries, case reports, and letters to the editors were also excluded. To identify all eligible articles, a search was conducted from inception to July 2022. PubMed, Ovid Embase, and African Journal Online were searched. The search strategy was jointly devised by the authors and is summarized in Table 1.

**Table 1:** Search strategy.

Database	MeSH terms/Keywords	Hits
PubMed	("external"[All Fields] OR "externally"[All Fields] OR "externals"[All Fields]) AND ("heart ventricles"[MeSH Terms] OR ("heart"[All Fields] AND "ventricles"[All Fields]) OR "heart ventricles"[All Fields] OR "ventricular"[All Fields] OR "ventricularization"[All Fields] OR "ventricularized"[All Fields]) AND ("drain s"[All Fields] OR "drainage"[MeSH Terms] OR "drainage"[All Fields] OR "drain"[All Fields] OR "drained"[All Fields] OR "draining"[All Fields] OR "drains"[All Fields])	2,275
Ovid Embase	External Ventricular Drain.mp	1, 658
African Journal Online	‘External ventricular drain’	41

The final search results were exported into Mendeley where duplicates were detected and removed after scrutiny. The deduplicated file was then exported into Rayyan.ai (a systematic review software where the rest of the screening took place). To ensure consistency, two authors screened each article at every stage. Where conflicts were apparent, they were resolved through consensus. Titles and abstracts were screened first, with emphasis on articles that specified EVD in African countries, while a full-text screening followed with a critical appraisal of the type of EVD used. Relevant data were extracted from the identified studies. The data included: the name of the study, year of publication, demographics, the country of study, the type of improvised EVD used, the way the improvised EVD was constructed, the indications for use, and the outcomes in terms of survival, and infection. Although the purpose of this study was to review the available literature on this topic, we conducted a limited meta-analysis of proportions to determine the combined incidence proportion of improvised EVD-related infections. A random-effects model was utilized to account for the heterogeneity of the studies, and it was carried out with the MetaXL add-on for Microsoft Excel with the use of a double arcsine transformation. We calculated the incidence proportion with a 95% confidence interval.

Results

**Figure 1:**
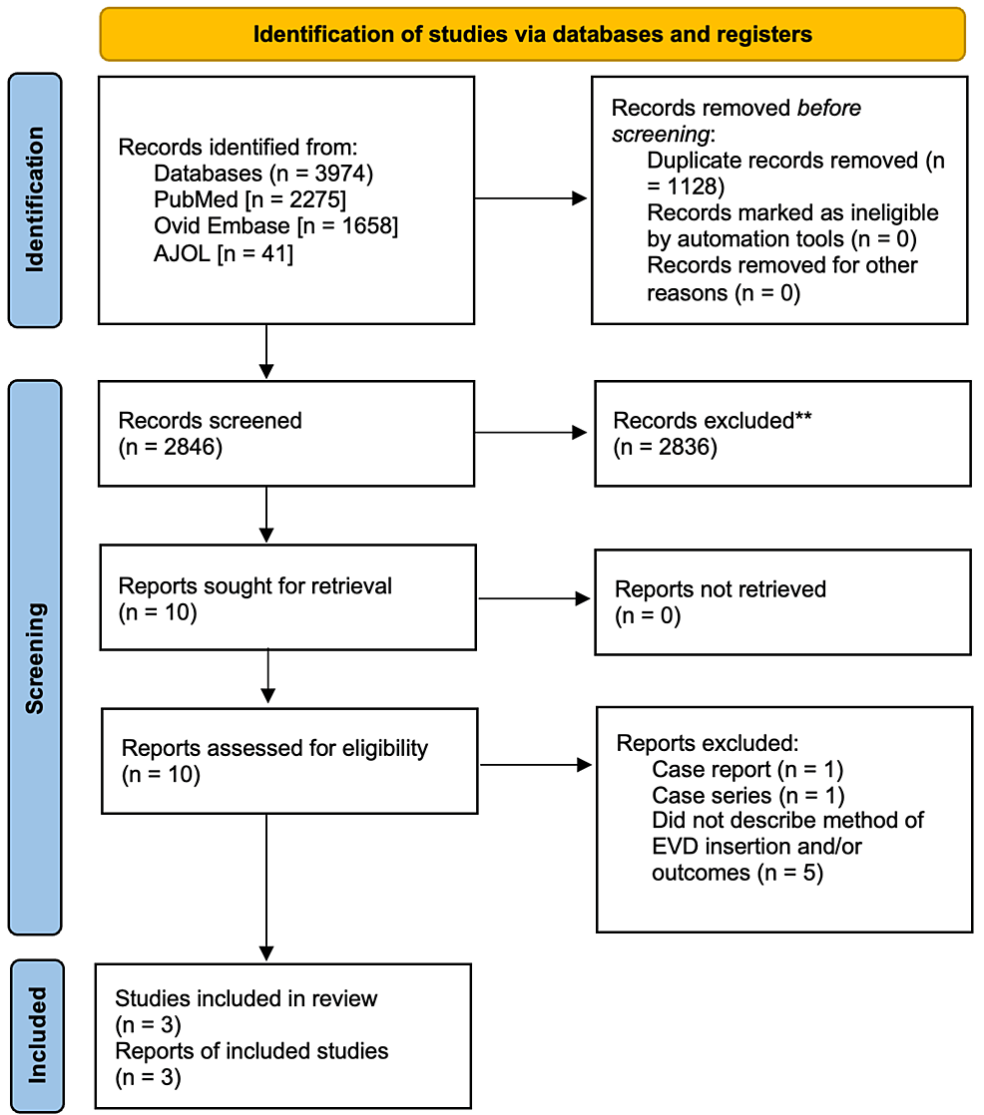
Prisma flowchart. EVD: external ventricular drain; AJOL: African Journal Online. The flowchart has been designed by Dr. Damilola Jesuyajolu.

The review and selection process is presented in the PRISMA flow diagram in Figure 1.

After the full-text screening of 10 manuscripts, seven studies were excluded. Reasons for exclusion included: a case report (1), case series (1), and not reporting the type/construction/outcome of the improvised EVD device (5). Three studies were identified and used [14-16]. The studies were conducted within the last decade and were from three different African countries (Nigeria, Tunisia, and Ethiopia). The studies involved 154 people from adult and pediatric populations. The summary of the study characteristics is seen in Table 2.

**Table 2:** Study characteristics. NA: not available

Study	Year	Country	Population	No of patients	%Male
Kammoun et al., 2018 [14]	2018	Tunisia	Mixed	33	NA
Ojo et al., 2015 [15]	2015	Nigeria	Pediatric	28	67.9%
Wondafrash and Tirsit, 2021 [16]	2021	Ethiopia	Mixed	93	55%

The comparison of the designs of the low-cost improvised EVDs can be seen in Tables 3-4.

**Table 3:** Parts of the proximal part of a standard EVD compared with low-cost alternatives used in some African centers. EVD: external ventricular drain; IV: intravenous fluid; NA: not available

Study	EVD catheter	Connecting tube	Proximal collecting system/ three-way stopcock	Connector tube to primary EVD system
Kammoun et al., 2018 [14]	14-gauge central catheter	Connector tube	3-way stopcock	Foley catheter
Kammoun et al .,2018 [14]	14-gauge central catheter	Coiled connector tube	NA	Redon tubing
Ojo et al., 2015 [15]	Sizes 6 or 8 (French gauge) feeding tube	IV fluid infusion set	NA	IV fluid infusion set
Wondafrash and Tirsit 2021 [16]	No. 6/8 sterile pediatric feeding tube	Connector	NA	NA

**Table 4:** Parts of the distal part of a standard EVD compared with low-cost alternatives used in some African centers. EVD: external ventricular drain; CSF: cerebrospinal fluid; EAM: external auditory meatus

Study	Distal collecting system/three-way stopcock	Adjustable EVD set for pressure measurement (or alternative)	Graduated burette for measuring CSF outflow (with outflow stopcock and outflow tubing)	Collection bag
Kammoun et al., 2018 [14]	NA	Manual reference measurement from the EAM	Graduated sterile urimeter (plastic part)	Graduated sterile urimeter (attached collection bag)
Kammoun et al., 2018 [14]	NA	Transfusion drip chamber placed against a flat transparent ruler all hung from a drip stand	Devacuumed graduated Redon bottle	Devacuumed graduated Redon bottle
Ojo et al., 2015 [15]	NA	Manual reference measurement from the EAM	Graduated sterile urine collection bag	Graduated sterile urine collection bag
Wondafrash and Tirsit, 2021 [16]	NA	Manual reference measurement from the EAM	Graduated sterile urine collection bag	Graduated sterile urine collection bag

**Figure 2:**
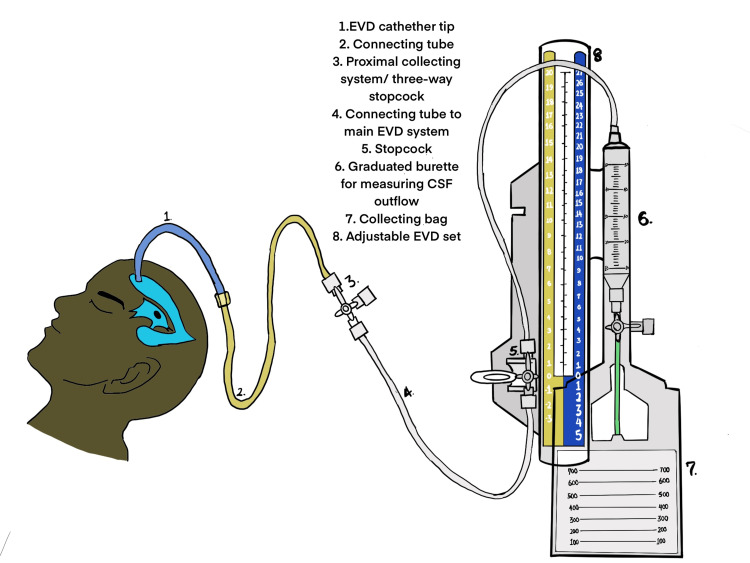
A schematic of the standard EVD device. EVD: external ventricular drain. The figure has been drawn by Dr. Damilola Jesuyajolu.

The standard EVD design can be seen in Figure 2.

The standard ventricular catheter was replaced by cheaper alternatives (a size 6/8 feeding tube or a 14-gauge central line catheter). This ventricular catheter was then manually connected to a connecting tube. The connecting tube had low-cost alternatives, and in the case of Ojo et al., was replaced by a fluid infusion set [15]. This formed the proximal part of the EVD device. The choice of the connecting tube that connects the graduated chamber to the primary EVD system varied between studies, with Kammoun et al. using alternatives like a manually redesigned Foley catheter or Redon tubing [14]. Setting the pressure involved a manual reference measurement done from the external auditory meatus (EAM). In one of the model designs reported by Kammoun et al., a repurposed blood transfusion drip chamber was placed against a transparent ruler mimicking the standard pressure measurement in a traditional EVD device [14].

Low-cost replacements for the graduated burette for measuring CSF outflow included a basic urine collection bag and a slightly more advanced urimeter bag. A standard urimeter bag has a measuring chamber that can be opened to let CSF flow into a collection bag. None of the improvised devices had provision for a distal stop cock. In Wondafrash and Tirsit’s study, posterior fossa tumors accounted for 46.2% of the indications for improvised EVD placement followed by supra-tentorial tumors (20.4%) and other indications like cerebellar stroke and/or hemorrhage [16]. Ojo et al. had similar indications [15]. The study reported indications that included: posterior fossa tumors (for surgery), hydrocephalus with hemorrhagic CSF (23.3%), ventriculitis with associated hydrocephalus, infected CSF (30%), and stroke with associated intraventricular hemorrhage + intracerebral hemorrhage. Aggregated outcomes from the three identified studies show that just over half of the sample survived post-EVD insertion (54%). The combined incidence proportion of EVD-related infections was 24%.

Discussion

We have been able to describe the low-cost alternatives to the standard EVD that are being used in African countries. We have also described the methods by which these devices are made while comparing them to the different parts of the standard EVD device. With the use of feeding tubes, infusion sets, urimeters, and catheter bags, functional low-cost EVD sets can be created. Our study, however, quotes infection rates as high as 24%. This figure is significantly higher than figures quoted in literature from centers in first-world countries that utilized standard EVD devices. These ranged between 3 and 10% [17-21]. The reason for the high infection rates may be related to the design of the improvised drains themselves. The surgeons often have to cut and repurpose other devices to fit the purpose of the improvised drains and this process may serve as a source of microbial contamination. Furthermore, these devices are not antibiotic-impregnated and lack filters which serve as barriers to colonization and infection. Although the EVD-related infection rate is significant, the absence of an EVD insertion, which is often the alternative, will likely increase the risk of mortality as these drains are inserted as life-saving interventions.

To the best of our knowledge, there has been no comprehensive review study looking at the use of improvised EVDs in Africa. The implications of these findings are critical for global neurosurgical care. With appropriate dissemination of the knowledge of how to make low-cost EVDs in low-resource settings, neurosurgical care can be effectively improved at a minimal cost. Rather than wait for EVD devices given as donations, or burden the patients with searching for EVD devices to buy as a result of a lack of supply from the hospitals, surgeons can easily utilize these devices that are readily available to them. Furthermore, there is a paucity of neurosurgeons in certain parts of Africa, and general surgeons and non-physicians are already being trained to deliver emergency neurosurgical services [22-25]. Incorporating the knowledge of making improvised external ventricular devices will go a long way to improving service delivery.

Although the benefits of these devices are obvious, there are still unanswered questions that require further studies, and areas to work on to reduce the rates of infection. Standard EVDs are often antibiotic-impregnated, which helps to prevent infection [26]. These improvised EVDs are plain, and research needs to be done to see how these low-resource alternatives can be improved in terms of infection control such as with antibiotic-impregnated EVD catheters. Retrieving CSF samples from standard EVD drains can be easily achieved with the use of stopcocks and sterile outlets; however, improvised devices do not have this luxury. Improvements need to be made in this regard. Despite the disadvantages of the improvised devices, the benefits are remarkable, considering the alternative of no intervention and the high associated risk of death. As these devices are used more frequently, and more reports are published, there will be an improvement in the quality and concurrent reduction in the associated complications. Finally, in this scoping review, we have identified a gap in the literature concerning this subject. There is a likelihood that more of these devices are being used in African countries but the outcomes and experiences have not been published. More must be done to ensure that data surrounding the use of these devices are published.

Despite our attempt at a comprehensive review, we had some limitations. In our review, we excluded articles that were not in the English language, which could have prevented us from including potentially relevant articles. There was a remarkable paucity of published studies, which in turn led to small sample size. The examined studies were not exhaustive, as we were not able to extensively investigate the factors that could be responsible for EVD-related infections. The paucity of data from these studies did not allow us to perform extensive statistical analysis, which could have further improved this study. Although we wanted to report our findings from the African perspective, limiting our findings to the continent may have limited the number of eligible studies available to us. The next step would be to expand our study to include other low- and low-middle-income countries as the body of literature on this subject grows. At the moment, this finding would be a significant addition to the existing literature.

## Conclusions

This study describes the experience of African centers with an improvised version of the EVD devices and their outcomes. It highlights how these devices are made. Although there has been a success with their use, the infection rate in this study was 24%. This will serve as a baseline for more research into the use of improvised EVD devices in low-resource settings, and a discussion as to how improvements can be made.
